# Recent Understanding on Diagnosis and Management of Central Nervous System Vasculitis in Children

**DOI:** 10.1155/2012/698327

**Published:** 2012-09-12

**Authors:** Ludovico Iannetti, Roberta Zito, Simone Bruschi, Laura Papetti, Fiorenza Ulgiati, Francesco Nicita, Francesca Del Balzo, Alberto Spalice

**Affiliations:** ^1^Department of Ophthalmology, Ocular Immunovirology Service, Sapienza University of Rome, Viale del Policlinico 155, 00161 Rome, Italy; ^2^Department of Pediatrics, Child Neurology Division, Sapienza University of Rome, 00185 Rome, Italy

## Abstract

Central nervous system vasculitides in children may develop as a primary condition or secondary to an underlying systemic disease. Many vasculitides affect both adults and children, while some others occur almost exclusively in childhood. Patients usually present with systemic symptoms with single or multiorgan dysfunction. The involvement of central nervous system in childhood is not frequent and it occurs more often as a feature of subtypes like childhood polyarteritis nodosa, Kawasaki disease, Henoch Schönlein purpura, and Bechet disease. Primary angiitis of the central nervous system of childhood is a reversible cause of severe neurological impairment, including acute ischemic stroke, intractable seizures, and cognitive decline. The first line therapy of CNS vasculitides is mainly based on corticosteroids and immunosuppressor drugs. Other strategies include plasmapheresis, immunoglobulins, and biologic drugs. This paper discusses on current understanding of most frequent primary and secondary central nervous system vasculitides in children including a tailored-diagnostic approach and new evidence regarding treatment.

## 1. Introduction

Vasculitides constitute a heterogeneous group of diseases characterized by inflammation of the blood vessel wall. Central nervous system (CNS) vasculitides in children may develop as a primary condition or secondary to an underlying systemic disease including infections, collagen vascular diseases, systemic vasculitides, and malignancies (Table 1) [1].

Although many vasculitides affect both adults and children, some, such as Kawasaki disease, occur almost exclusively in childhood. Other vasculitides (e.g., temporal arteritis) rarely if ever occur in childhood, and others, like polyarteritis and polyangiitis GPG (also known as Wegener's granulomatosis) have different etiological, clinical, and prognostic characteristics in children [2]. For these reasons the EULAR (European League against Rheumatism) recently proposed the new consensus criteria for the classification of childhood vasculitides (Table 2) [2]. 

When CNS is involved in the course of vasculitides children may present with intractable seizures, cranial nerve deficits, paresis, and/or cognitive deficits. The diagnosis of CNS vasculitides is particularly difficult because the available investigative modalities have limited sensitivities and specificities. The most helpful diagnostic tests include cerebrospinal fluid analysis, MRI (MR angiography/venography (MRA/MRV) of the brain, and angiography. In particular conventional angiography remains the gold standard for identifying lesions in cerebral vasculitis due to missed lesions with MRA, particularly in younger patients. Conventional angiography is more sensitive than even high-quality MRA at detecting involvement of the posterior circulation and distal cerebral vessels. To date MRA is a reasonable initial modality in the investigation of suspected CNS vasculitis but in cases of abnormal parenchymal MRI and normal MRA, CA should be considered [3]. However, brain biopsy may be required to diagnose small vessel vasculitides. The differential diagnosis includes a wide range of conditions, such as degenerative vasopathies, embolic diseases, or coagulation disorders (Table 3) [1]. 

**Figure 1:**
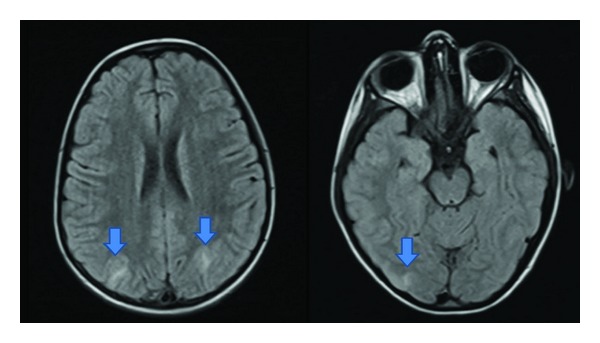
MRI Axial T2-Flair image. A six-years girl with HSP complicated by PRESS. Bilateral hyperintense lesions of subcortical white matter in the occipital region.

Different treatments have been proposed including corticosteroids, immunosuppressive agents, such as methotrexate and cyclophosphamide, and intravenous gammaglobulines to induce or maintain remission and to prevent long-term vascular complications (Figure 1). In addition to a specific therapy that can vary depending on the nature of vasculitis, patients with cerebral involvement also need an acute symptomatic therapy which varies depending on the specific clinical manifestation (stroke, headache, epilepsy, etc.). This acute symptomatic treatment must be undertaken immediately, well before the precise etiologic diagnosis. Supportive therapy for all types of CNS vasculitis may include anticonvulsants for children with seizures, and antipsychotic agents for those with hallucinations or severe behavior difficulties. Children who are treated with high-dose corticosteroids may require ranitidine or a proton pump inhibitor to treat associated gastritis and calcium and vitamin D supplementation to preserve their bone health.

In this paper, we provide an update description of different immunointerventions in vasculitides, comparing and discussing current literature about practical management of childhood CNS vasculitides.

## 2. Childhood Polyarteritis Nodosa

Childhood polyarteritis nodosa (PAN) is a necrotizing vasculitis, affecting medium size blood vessels. PAN includes two different subtypes, the classical systemic form presenting with a wide range of clinical manifestations including dermatologic, musculoskeletal, nervous, renal, and gastrointestinal systems and the more frequent cutaneous form (CPAN) that involves only the skin. Since the aim of this paper is to describe the treatment options in vasculitis with brain involvement, in this section will refer only to the systemic form.

The diagnosis of PAN requires the evidence of necrotizing vasculitis or angiographic abnormalities of medium-/small-sized arteries (mandatory criterion) plus one of five criteria: (1) skin involvement; (2) myalgia/muscle tenderness; (3) hypertension; (4) peripheral neuropathy; (5) renal involvement [2].

Neurological involvement has been reported in about 50% of patients, presenting with peripheral nervous system involvement (mainly paresthesias and polyneuropathies) more frequently than CNS involvement (including encephalitis, convulsions, hemiparesis, subarachnoid hemorrhage, and isolated cranial nerve palsies) [4]. Myalgias are also very frequent neurological features. A combined biopsy of muscle usually and nerve demonstrates the necrotizing granulomatous inflammation [5]. Ischemic stroke, hemorrhages, and a progressive encephalopathy with or without seizures may occur [4]. Because of the rarity of systemic PAN in children, treatment options are mainly based on adult literature giving a central role to oral corticosteroid as first line treatment [4] and indicating second line therapies based on azathioprine, intravenous solu-Medrol, and cyclophosphamide. In children presenting with mild disease steroid alone may be recommended [6]. 

Hashimoto et al. reported a success rate 41.7% in the treatment of PAN with plasmapheresis [7]. In the series reported by Guillevin et al. plasma exchange have no added benefit to steroid ± cyclophosphamide [8].

Ozen, in her study, suggests a treatment schedule for severe disease based on prednisone 2 mg/kg/die to be tapered after clinical suppression. According to this author cyclophosphamide should be also given, orally, in a dose of 2 mg/kg/day for the first three months, after which azathioprine should be substituted and continued after a year [9], while Dillon and colleagues, in their educational review, also consider pulsed intravenous infusions for up to 6 months or for shorter periods if remission is achieved [6]. Maintenance therapy with daily or alternate-day low-dose prednisone and oral azathioprine is usually continued up to 18 months. Other maintenance agents include methotrexate and cyclosporin A. For those patients whose conditions are refractory to multiple immunosuppressive agents and corticosteroid dependent, oral mycophenolate mofetil, and infliximab may enable tapering and eventual discontinuation of the corticosteroids [4].

Concerning refractory cases, potential efficacy of intravenous Ig were reported in recent studies [10], while some other studies report successful outcomes using biologic agents as infliximab or rituximab [11]. Gianviti et al., discussed the possible benefit from the addition of plasma exchange to immunosuppressive medication in life-threatening situations, nevertheless still further trial are needed to resolve the question of this procedure's value in treating childhood vascular diseases [12].

## 3. Kawasaki Disease

Kawasaki disease (KD) is an acute febrile systemic vasculitis occurring in medium-sized vessels, especially coronary arteries. It affects mainly infants and children under 5 years of age and it is associated with the presence of coronary artery lesions (CALs) such as coronary artery dilatations and ectasias. CALs occur in almost 25% of untreated patients [13–16] and may develop into aneurismal formation, thrombotic occlusion and fistula formation or progress to ischemic heart disease and premature atherosclerosis [17]. The clinical presentation is characterized by prolonged fever, polymorphous skin rash, nonpurulent conjunctival injection, extremity changes, oral mucosal changes, and cervical lymphadenopathy. The CNS involvement occurs in about 0.4% of children in the form of aseptic meningitis, meningoencephalitis, hypoperfused brain, ischemia, cerebral and cerebellar infarction, and subdural effusion [18, 19]. In the case of aseptic meningitis we must distinguish two different forms, first one being secondary to the disease and second one following IVIG administration [20]. Clinical manifestations of CNS involvement include seizures and disturbance of consciousness [21]. Prognosis of neurological complications is generally good, although sequelae such as myoclonic seizures, hemiparesis, and moyamoya disease have been reported in a small percentage of patients [18]. Although MRI scans revealed no abnormalities at the acute stage of the disease, the CNS manifestations associated with KD might be due to focal impairment of blood flow caused by cerebral vasculitis. In six of 21 children with acute Kawasaki disease, single-photon emission computed tomography (SPECT) imaging demonstrated localized cerebral hypoperfusion without neurologic findings [22].

Standard therapy is intravenous immunoglobulin plus aspirin which showed a good outcome in most of the patients, suggesting that proinflammatory cytokines are responsible for the progression of the disease, and that removal of these cytokines from the circulation will be a major strategy of treatment [14, 23]. Randomized controlled trials have shown that a single infusion of 2 g/kg of IVIG given 5–10 days after the onset of fever, eliminated fever in 85–90% of children within 36 h and significantly reduced the risk of CALs [24]. Five possible mechanisms include Fc receptor blockade, neutralization of the causative agents, or a toxin produced by an infectious agent, an immunomodulating effect, induction of suppressor activity, and modulation of the production of cytokines and cytokine antagonists [25]. 

Aspirin remains one of the mainstays of therapy because of its antiinflammatory and anti-thrombotic actions [26]. During the acute phase of illness, aspirin is administered at anti-inflammatory doses with IVIG. Still controversial is the duration of high dose aspirin administration, which varies between different centers. High-dose aspirin and IVIG appear to possess an additive anti-inflammatory effect [13]. Discontinuation of high-dose aspirin is usually recommended when patient is afebrile, switching to low-dose aspirin (3–5 mg/kg/die) until there is no evidence of coronary artery lesions and inflammatory markers have returned to normal levels (usually 6–8 weeks after disease onset). Nevertheless, recent studies suggest that children exposure to high dose aspirin therapy in the acute phase of KD is unnecessary, claiming that available data do not show appreciable benefit to IVIG therapy and clinical resolution [13]. 

Other therapeutic options (in addition to retreatment with immunoglubulins 1-2 g/kg) have been proposed for refractory KD: pulsed corticosteroid treatment (intravenous methylprednisolone 30 mg/kg per day for 3 days), infliximab, abciximab, plasmapheresis, plasma exchange, and immunosuppressants such as Cyclophosphamide and Cyclosporin A [13, 27–31]. The TNF alpha blockers (such as infliximab) and platelet glycoprotein Ibis/Imia receptor inhibitors (such as abciximab), seem to benefit KD patients, especially those patients/cases refractory to IVIG and those patients who developed aneurysms. Concerning infliximab administration, it is currently increasing for KD patients with recrudescent fever or persistently elevated inflammatory markers to prevent coronary aneurysm and stricture formation and a dose of 4.8 mg/kg, diluted in 250 mL of normal saline, may be suggested [29, 32–35]. Instead, abciximab administration is commonly used to prevent thrombosis and to decrease aneurysm size in patients with large coronary artery aneurysms [36]. A recent open label trial have been performed by Choweiter et al. to determine safety of Etanercept as adjunctive therapy for the treatment of coronary artery lesions in KD and data obtained support the performance of larger efficacy trials for etanercept use in pediatric patients [37]. However, treatment with TNF-*α* antagonists in the patients with KD does raise some safety concerns. These include a potential for myocarditis, and development of coronary artery abnormalities, ischemia and the risk of TB and cancer (mainly lymphomas in patients receiving etanercept) although the existence of a cause and effect relationship between anti-TNF use and lymphomas remains controversial [37].

Recent studies have shown that statin therapy seems to significantly improve chronic vascular inflammation and endothelial dysfunction in children with KD, but still further study is needed to determine the safety and efficacy of statins in children [38].

## 4. Henoch Schönlein Purpura

Henoch Schönlein purpura (HSP) is a systemic IgA-mediated vasculitis affecting predominantly small blood vessels. It is the most common form of small vessel vasculitis in children [39]. The pathogenesis of HSP remains unknown; however, HSP is generally believed to be an immune complex-mediated disease characterized by the presence of polymeric IgA1 (pIgA1)-containing immune complexes predominantly in the dermal, gastrointestinal and glomerular capillaries [40].

HSP is a multiorgan system disease and its major manifestations include cutaneous purpura, arthalgia, enteritis, and nephritis [41]. Clinical criteria for HSP according to EULAR include (1) purpura (mandatory criterion) or petechiae, with lower limb predominance, (not related to thrombocytopenia); (2) abdominal pain (may intussusception and gastrointestinal bleeding); (3) histological changes showing leucocytoclastic vasculitis with predominant IgA deposit or proliferative glomerulonephritis with predominant IgA deposit; (4) arthritis or arthralgias; (5) renal involvement with hematuria and/or proteinuria. A patient meets the classification of HSP, if at least 2 of 4 criteria are present [42].

Pulmonary, cardiac, or genitourinary complications occur rarely, as well as neurological manifestations. The CNS involvement has been reported in 1–8% of children. Possible neurological presentations include headache, altered level of consciousness, seizures, focal neurological deficits, visual abnormalities and verbal disability, peripheral neuropathy, and facial palsy [43, 44]. Imaging studies (MRI or CT scan) might reveal lesions suggestive of small vessel vasculitis as ischemic vascular lesions almost always involving two or more vessels, intracerebral haemorrhages, diffuse (mainly posterior) brain edema, or thrombosis of the superior sagittal sinus [45, 46]. Posterior reversible encephalopathy syndrome (PRES) has been described in children with HSP [3]. The pathogenesis of the PRES in HSP is not exactly clear, although two possible mechanisms have been considered. The first regards hemodynamic change ascribable to severe hypertension and renal insufficiency that may complicate HSP [47]. Since the vertebrobasilar and posterior cerebral arteries are sparsely innervated by sympathetic nerves, severe hypertension can easily impair autoregulation of the blood pressure in their perfusion areas, sometimes causing RPLS characterized by vascular edema due to damage to the blood-brain barrier [48]. Encephalopathy can develop in HSP even without severe hypertension and renal insufficiency [49], and in these cases CNS vasculitis is suspected as the likely pathogenetic mechanism, although this hypothesis remains unproven by histopathology of the brain [47].

Pharmacologic options include prednisone, immunosuppressive drugs, warfarin, and dipyridamole. Not all HSP patients need early steroid and/or immunosuppressors treatment, and treatment should be targeted at patients who have a high risk of renal involvement or severe extrarenal symptoms [50]. The extrarenal manifestations of HSP are managed by appropriate symptomatic measures. 

Severe skin lesions may require oral corticosteroids, which may also improve abdominal pain and protein-losing enteropathy. Severe gastrointestinal complications may occasionally require surgical intervention [50]. 

The start of therapy in children at risk of renal complications may also reduce the risk of cerebral complications when you consider that renal hypertension is one of the most well-known risk factors for CNS involvement in children with HSP.

Prednisone is generally used at dose of 1 mg/kg/day for 2 weeks, with weaning over the subsequent 2 weeks. Unlike previous data [51] a randomized-double-blind-placebo-controlled trial showed that early prednisone treatment did not prevent the development of renal symptoms, but prednisone was definitely effective in altering the course of renal disease in patients with signs of mild renal symptoms at inclusion or within the first month after the diagnosis [52].

Recently Garzoni et al. suggested that CNS dysfunction in HSP results from a vascular obstruction, from an intracerebral haemorrhage or from severe hypertension. In this light the author suggests that like in adults with stroke, the initial management of patients with suspected cerebral HSP includes control of arterial hypertension, seizures, and repair of disordered hemostasis. In patients with intracerebral haemorrhage, the indications for surgery are controversial and vary with the site and the size of the bleed. Like in severe HS glomerulonephritis, combined therapy with corticoids and cyclophosphamide is appropriate in a patient with relevant ischemic cerebral lesions and HSP, instead it is considered not necessary in those patients with peripheral or cranial neuropathy, which spontaneously tends to recovery [45]. In cases which are complicated by Guillain Barré Syndrome the treatment with intravenous immunoglobulin (1 g/kg/die) or plasma exchange are suggested [4]. Furthermore, recent studies propose leukocytapheresis for treatment of patients with HSP refractory to both steroid and immunoglobulin therapy, aiming to the removal of proinflammatory cytokines produced by activated inflammatory cells [53].

## 5. Behcet Disease

Behcet disease (BD) is a multisystemic chronic relapsing vasculitis characterized by recurrent mucoutaneous lesions, ocular and vascular involvement. Clinical criteria for diagnosis of BD include oral ulcers at least three times in 12 months and any two of the following: recurring genital sores/ulcers, eye inflammation with loss of vision, characteristic skin lesions, or positive pathergy (skin prick test) [54].

The involvement of CNS occurs in 11% to 50% of pediatric patients, with parenchymal and nonparenchymal manifestations [55]. Neuro-Behcet's disease (NBD) is defined as evidence of Behcet's disease plus neurologic involvement, not explained by other conditions or exposures. The most common manifestations of NBD are cranial nerve palsy, dysarthria, unilateral, or bilateral pyramidal tract signs, ataxia, consciousness disturbance. Less common CNS manifestations include hemiparesis, cognitive-behavioral changes, emotional changes, extrapyramidal signs, and seizures [56]. Parenchymal lesions on cranial magnetic resonance imaging may involve brainstem, hemispheric white matter, cerebellum, spinal cord, and/or leptomeninges while nonparenchymal lesions may include dural sinus thrombosis, pseudotumor cerebri, arterial occlusion, and aneurisms [55]. The treatment of NBD is still controversial: the corticosteroids (starting with intravenous steroids such as methylprednisolone 500 mg–1 g/day for 1 to 3 days and then switching to oral prednisone 0.8 mg/kg/day [55, 57]) and disease modifying antirheumatic drugs such as azathioprine (1-2 mg/kg orally every day [55]) and methotrexate are conventionally used as first line agents [58], while cyclophosphamide treatment is usually reserved to high-risk patients [57]. 

In particular, concerning neurological involvement, suggested therapeutic approach, according to EULAR's recommendations [59], include corticosteroids for dural sinus thrombosis (brief courses of corticosteroids if presenting with increased intracranial pressure and headaches), and for parenchymal involvement. In this case high doses of pulsed corticosteroids, usually 3–7 pulses of intravenous methylprednisolone 1 mg/day, may be given during attacks, followed by maintenance oral corticosteroids tapered over 2-3 months. Immunosuppressive drugs may also be given to prevent recurrences and progression. Studies on methotrexate suggest beneficial effects while chlorambucil is rarely used due to high risk of serious adverse effects (such as myelotoxicity and increased risk of malignancies) preferring azathioprine instead (2.5 mg/kg/day or in more severe cases monthly pulses of cyclophosphamide). Concerning resistant cases, agents to be tried may include IFN*α* and TNF*α* antagonists [59–64]. In fact, recent studies have suggested that the use of biologic drugs, such as anti-TNF antibodies (infliximab) could cause downstream effects on cerebrospinal fluid interleukin-6 [55, 65]. Up to the present this promising therapeutic approach, was only reported to be effective in adult patients with severe ocular and various extraocular manifestations, including central nervous system involvement, but not yet in pediatric patients. Adalimumab, which has a mechanism of action similar to that of infliximab, has been also suggested as an alternative therapy with a favorable side-effect profile for paediatric patients with acute NBD [55]. The promising results with Etanercept therapy in juvenile-onset BD patients, characterized by refractory multiorgan involvement, were also presented by Cantarin and colleagues [66]. Recent studies have stressed the central proinflammatory role of IL-6 in “in situ” evolution of NBD [67], suggesting IL-6 inhibition as a new therapeutic strategy for this disease. 

## 6. Childhood Primary CNS Vasculitis

Primary angiitis of the central nervous system in children (c-PACNS), represent a group of idiopathic vasculitis, involving either medium/large vessels or small vessels and causing neurological deficits and deterioration [1, 68]. 

There are two types of c-PACNS: medium-large vessel and small vessel vasculitis. Medium-large vessel disease affects arteries that are large enough to be differentiated by conventional angiography. In patients with small vessel childhood primary angiitis of the CNS (SVcPACNS), angiography findings are typically negative and thus diagnosis must be confirmed by brain biopsy [68].

Recently it has been underlined that when SVcPACNS is suspected in a child (uncertain cases where children present with acquired neurological deficits, or abnormal inflammatory markers or CSF analysis, with or without an abnormal MRI) a brain biopsy should be performed. Lesional biopsies are preferred; however, nonlesional biopsies may succeed in yielding the diagnosis [69]. Vasculitides of the large-to-medium vessels (or angiography-positive cPACNS) are associated with headache, hemiparesis, hemisensory deficits and fine motor deficits [70]. Intractable seizures, cognitive decline, behavioural changes, and systemic features, such as fever and malaise, tend to be associated more frequently with small-vessel inflammation [6].

The diagnosis of PACNS may be considered with symptoms of a multifocal or diffuse CNS disorder with remitting or progressive course, cerebrospinal fluid (CSF), and MRI findings supporting the diagnosis of vasculitis, and finally either an angiography with a vasculitic pattern or a leptomeningeal and parenchymatous biopsy proving vasculitis [71]. 

Calabrese et al., proposed the following preliminary clinical criteria for PACNS in adults: (1) an acquired neurologic deficit that remains unexplained after a thorough initial basic evaluation, (2) either classic angiographic or histopathologic features of angiitis within the CNS, and (3) no evidence of systemic vasculitis or any other condition to which the angiographic or pathologic features could be secondary. Most reported pediatric cases fit these criteria [71]. 

There is a paucity of data regarding the efficacy of immunosuppressive treatment of PACNS both in adults and children. 

Some may argue that benign angiopathy of the CNS or transient cerebral arteriopathy needs no immunosuppressive treatment [71] or a relatively short course of corticosteroids (<6 months) [72]. It has recently been proposed the term reversible cerebral vasoconstriction syndrome (RCVS) instead benign angiopathy, referring to a group of disorders characterized by acute onset of headaches, with or without neurologic deficits, and prolonged but reversible cerebral vasoconstriction. Although RCVS can mimic PACNS it is a noninflammatory vasospastic disease primarily interesting adults, with different therapeutic and prognostic implications [73]. In RCVS, successful treatment has been reported with calcium channel blockers, short-term glucocorticoids, and magnesium sulphate [74]. 

Particular attention should be given to exclusion of systemic infection resembling cPACNS, such as spirochetal, rickettsial, and viral diseases as well as bacterial endocarditis, before giving cyclophosphamide.

Treatment recommendations in PACNS usually indicate two subsequent phases, first one including a combination of steroids and pulse cyclophosphamide (induction phase, during the first 6 months) and second one with either methotrexate (MTX), azathioprine (AZA), or mycophenolate mofetil (MMF) (maintenance phase, during the following 1 or 2 years) [68, 75]. The use of antiplatelet or anticoagulant medications in the treatment for cPACNS has been controversial [1].

Sen et al., reported three children (two large-medium vessels cPACNS and one SVcPACNS) treated with steroids and cyclophosphamide in the induction phase and MTX or MMF or AZA. All the patients presented here initially received and responded to IV methylprednisolone followed by oral prednisolone that was gradually tapered. All patients relapsed while on either MTX or AZA and steroids, hence MMF was introduced. The authors concluded that MMF is considered as the maintenance immunosuppressive agent in the management of refractory cPACNS [75].

Recently a similar treatment protocol has been evaluated in SVcPACNS and it consisted of induction therapy with steroids (2 mg/kg daily weaned every 4 weeks to 50, 40, 30, 25, and 20 mg daily and then by 2.5 mg every 4 weeks until completed) and pulses of intravenous cyclophosphamide (Seven pulses of 500–750 mg/m² intravenous cyclophosphamide every 4 weeks with cotrimoxazole prophylaxis) followed by maintenance therapy (during 18 months) with either azathioprine (2-3 mg/kg) or mycophenolate mofetil (800–1200 mg/m² per day) [76]. 

Hutchinson et al. suggested that the mycophenolate mofetil maintenance therapy seems to cause less adverse events compared with azathioprine therapy [76]. Furthermore all patients should need calcium and vitamin D as well as bone protection agents and pneumocystis infection prophylaxis [77]. Supportive therapy for all types of CNS vasculitis may include anticonvulsants for children with seizures, and antipsychotic agents for those with hallucinations or severe behaviour difficulties. Children who are treated with high-dose corticosteroids may require ranitidine or a proton pump inhibitor to treat associated gastritis and calcium and vitamin D supplementation to preserve their bone health [1]. 

## 7. Conclusions 

The involvement of the CNS must always be considered in children with systemic vasculitis and neurological symptoms such as headache, alterated consciousness, seizures, and focal neurological deficits. The diagnosis of cerebral vasculitis is often a challenge for the physician as these may be confused with other disorders such as infectous disease and coagulation disorders. The first line therapy of CNS vasculitis is mainly based on corticosteroids and immunosoppressive drugs. Plasmapheresis and IVIg may have beneficial effects in the treatment of critical cases. Recently the possible role of biologic drugs in the treatment of vasculitis has been evaluated in adults. However there is a paucity of trials testing the efficacy and safety of these therapeutic strategies in children. 

## Figures and Tables

**Table 1:** Causes of secondary central nervous system vasculitis in children [1].

Infections	Viral: varicella, HIV, hepatitis C
	Bacterial: Lyme disease, tuberculosis
	Fungal
	Parasitic

Systemic vasculitis	Kawasaki disease
	Henoch-Schönlein purpura
	Polyarteritis nodosa
	Wegener granulomatosis
	Microscopic polyarteritis nodosa
	Takayasu arteritis

Systemic connective tissue diseases	Systemic lupus erythematosus
	Dermatomyositis
	Sjogren syndrome

Inflammatory bowel diseases	

Sarcoidosis	

Vascular injury	Dissection
	Radiation

Drugs	Amphetamines
	Contraceptives

Neoplasms	

Graft versus host diseases	

**Table 2:** New classification of childhood vasculitis [2].

Predominantly large vessel vasculitis	Takayasu arteritis
Predominantly medium-sized vessel vasculitis	Childhood polyarteritis nodosa
	Cutaneous polyarteritis
	Kawasaki disease

Predominantly small vessels vasculitis	(A) Granulomatous:
	Wegener's granulomatosis
	Churg-Strauss syndrome
	(B) Nongranulomatous:
	Microscopic polyangiitis
	Henoch-Schönlein purpura
	Isolated cutaneous leucocytoclastic vasculitis
	Hypocomplementic urticarial vasculitis

Other vasculitis	Behcet disease
	Vasculitis secondary to infection (including hepatitis B associated polyarteritis nodosa), malignancies, and drugs, including hypersensitivity vasculitis
	Vasculitis associated with connective tissue diseases
	Isolated vasculitis of the central nervous system
	Cogan syndrome
	Unclassified

**Table 3:** Differential diagnosis of CNS vasculitides in children.

Infectous disease	Viral/bacterial encephalitis
	Viral/bacterial menigitidis
	Viral/bacterial sepsi

Metabolic disease	Mitochondrial disease
	Amino acid disorders
	Organic acidemias
	Urea cycle disorders
	Fatty acid oxidation disorders
	Fabry disease
	Homocystinuria
	Leukodystrophies

Demyelinating diseases	Multiple sclerosis
	Acute-disseminated encephalomyelitis

Thromboembolic diseases	Antiphospholipid syndrome
	Hypercoagulability states
	Cholesterol embolisms
	Cardiac myxoma
	Nonbacterial thrombotic endocarditis

Malignancies	Multifocal glioma
	CNS lymphoma
	Angiocentric lymphoma
	Intravascular lymphoma (malignant angioendotheliomatosis)

Autoimmune disease	Celiac disease
	Hashimoto's encephalitis
	Sarcoidosis
	Systemic lupus erythematosus
	Neuronal antibody associated (NMDA-receptor associated encephalitis)

Vascular	Arterial dissection
	Fibromuscular dysplasia
	Moyamoya disease
	Vasospastic disorders

Nutritional	Vitamin B12 deficiency
